# The Effects of Repetitive Transcranial Magnetic Stimulation on Gait, Motor Function, and Balance in Parkinson’s Disease: A Systematic Review and Meta-Analysis of Randomized Controlled Trials

**DOI:** 10.3390/jcm15010166

**Published:** 2025-12-25

**Authors:** Myoung-Ho Lee, Ju-Hak Kim, Je-Seung Han, Myoung-Kwon Kim

**Affiliations:** 1 Department of Physical Therapy, Graduate School, Daegu University, Jillyang, Gyeongsan 38453, Gyeongbuk, Republic of Korea; hotayaaa@gmail.com (M.-H.L.); rlawngkr2000@naver.com (J.-H.K.); hanjs0618@naver.com (J.-S.H.); 2 Department of Physical Therapy, College of Rehabilitation Sciences, Daegu University, Jillyang, Gyeongsan 38453, Gyeongbuk, Republic of Korea

**Keywords:** balance, gait, motor function, Parkinson’s disease, repetitive transcranial magnetic stimulation

## Abstract

**Objective**: This study aimed to systematically evaluate the therapeutic effects of repetitive transcranial magnetic stimulation (rTMS) on gait, motor function, and balance in patients with Parkinson’s disease (PD) and identify optimal stimulation parameters for clinical application. **Methods**: This systematic review and meta-analysis of randomized controlled trials (CTs) was conducted according to the Preferred Reporting Items for Systematic Reviews and Meta-Analyses (PRISMA) guidelines. PubMed, EMBASE, Cochrane Central, Scopus, and Ovid-LWW were searched until December 2024 for RCTs evaluating the effects of rTMS on PD-related gait, balance, or motor outcomes. Nineteen studies (*n* = 547) met the inclusion criteria. Data on study characteristics, rTMS protocols (frequency, target area, pulses, session duration, number of sessions, and treatment duration), and outcome measures (freezing of gait questionnaire [FOG-Q], gait speed, Unified Parkinson’s Disease Rating Scale Part III [UPDRS-III], UPDRS total, and timed up and go [TUG] test) were extracted. Effect sizes (Hedges’ g) were pooled using inverse variance meta-analysis, heterogeneity was assessed using I^2^, and publication bias was assessed using funnel plots and Egger’s regression. **Results**: rTMS produced significant improvements in gait freezing (FOG-Q: g = −0.74; 95% confidence interval [CI] [−1.05, −0.43]; *p* < 0.001), gait speed (g = 0.62; 95% CI [0.29, 0.95]; *p* < 0.001), and motor symptoms (UPDRS-III: g = −0.42; 95% CI [−0.70, −0.15]; *p* = 0.003). No significant effects were observed for UPDRS total (g = 0.18; *p* = 0.58) or balance (TUG, g = −0.29; *p* = 0.06). Egger’s test indicated publication bias for gait speed (*p* = 0.016); however, trim-and-fill imputed zero studies. Subgroup analyses indicated that high-frequency stimulation of the supplementary motor area (SMA) for ≥20 min over 10 sessions (total duration <2 weeks or ≥2 weeks) optimally improved gait speed, whereas low-frequency stimulation targeting M1 and SMA with >1000 pulses per session for 20 min over 10 sessions within <2 weeks most effectively improved the UPDRS-III scores. **Conclusions**: rTMS exerts moderate and significant benefits on gait and motor performance in PD, particularly when tailored protocols involving SMA or M1 stimulation are employed. High-frequency SMA protocols improve gait speed, whereas low-frequency M1/SMA protocols optimize motor symptom relief. These findings provide evidence-based guidance for rTMS implementation in PD rehabilitation.

## 1. Introduction

Parkinson’s disease (PD) affects 6–10 million people worldwide and is the second most prevalent neurodegenerative disease after Alzheimer’s dementia. Its prevalence is increasing in aging societies [1,2]. PD is characterized by resting tremors, bradykinesia, gait disturbances, rigidity, and postural instability, which significantly impair daily living [3]. As PD progresses, physical and psychological disabilities worsen, eventually rendering independent living impossible [4]. Consequently, medical costs for the treatment of PD have increased, placing a growing socioeconomic burden on society [5].

One of the hallmark features of gait disturbance in patients with PD is freezing of gait (FOG), where the feet suddenly become temporarily immobile. As the disease progresses, the frequency and duration of FOG episodes tend to increase [6]. Approximately 50% of patients with >10 years of disease experience FOG [7], and approximately 80% of all patients with PD report this symptom [8]. It particularly occurs when initiating or stopping walking, turning in narrow spaces, performing complex tasks, or reaching a destination and presents as if the feet are glued to the ground. This symptom leads to a loss of balance and is a major cause of falls [6,9]. FOG-induced falls in patients with PD are associated with muscle strength, flexibility, postural instability, balance, and cognitive impairments [10]. Balance disorders in PD arise from a complex interaction of sensory deficits, impaired muscle responses, and postural control issues [11]. Stable balance depends on the integration of visual, vestibular, and proprioceptive inputs; however, dysfunction in these systems hinders maintaining postural stability [12]. Furthermore, sudden perturbations in PD disturb postural control, with poor muscle timing, excessive contraction, and rigidity contributing to balance problems [13].

Among the methods of treating PD, pharmacological therapy is highly effective in the early stages when motor symptoms first arise. However, in later stages, medication alone becomes insufficient, and it requires combination with other treatment modalities [14]. Surgical interventions can improve motor symptoms; however, they are often associated with side effects and economic burden [15]. Noninvasive rehabilitation techniques aim to enhance functional capacity and minimize secondary complications by focusing on posture, motor function, balance, gait training, and physical conditioning. These approaches include strategies such as external cueing to facilitate automatic motor responses and balance-oriented training to prevent falls and improve postural control [16,17,18]. However, the need for long-term rehabilitation limits their effectiveness unless combined with other therapies.

Another noninvasive method is repetitive transcranial magnetic stimulation (rTMS) [19]. rTMS is a noninvasive neuromodulatory technique that applies rapidly changing magnetic fields to the scalp, inducing electric currents in targeted cortical regions. Depending on the stimulation parameters, rTMS can increase or decrease cortical excitability and has been investigated as a potential intervention for improving gait and motor symptoms in PD. The stimulation induces either excitation or inhibition of specific brain regions by generating depolarization and subsequent action potentials. rTMS protocols are often differentiated by frequency (e.g., high frequency > 5 Hz for excitation, low frequency ≤ 1 Hz for inhibition) to induce and sustain excitability changes even after the stimulation ends [18]. A highly time-efficient, patterned form of rTMS is theta burst stimulation (TBS). TBS delivers short, high-frequency bursts repeated in a theta rhythm (5 Hz), which allows it to induce strong and long-lasting plasticity effects in a significantly shorter duration (typically < 5 min) compared to conventional rTMS protocols [20,21]. When targeted at brain areas responsible for gait or balance, related improvements may occur [22]. rTMS induces and sustains excitability changes; unlike other TMS pulses, it uses specific repeated frequencies to maintain effects even after the stimulation ends [19]. Therefore, a multidisciplinary approach involving both invasive and noninvasive methods is needed, depending on the patient’s condition and environment [23]. Although a multidisciplinary approach that integrates invasive and noninvasive strategies is often required for optimal rehabilitation, the clinical usefulness of rTMS is still not fully established because previous studies have yielded mixed results and have used highly heterogeneous stimulation protocols.

Randomized controlled trials (RCTs) on rTMS have investigated its effects on improving gait speed to alleviate FOG and its influence on balance and motor performance [24,25,26]. However, the target area for rTMS has varied across studies, and many studies have used small sample sizes. In addition, different perspectives and outcomes were reported, highlighting the need for integrated information. Although some meta-analyses on rTMS have reported significant findings in motor performance [25] or gait function [24], no study has comprehensively analyzed its effects on motor performance, gait, and balance. Some selected studies included nonrandomized trials or crossover designs. In addition, variations in the target area, frequency, sample size, and protocols limit the generalizability of results. Evaluating the effects of rTMS on motor symptoms in patients with PD is highly meaningful; however, a more precise and detailed analysis is needed. In summary, previous meta-analyses either focused selectively on specific outcome measures or included studies with heterogeneous designs, which limited the interpretability of their findings. Therefore, a comprehensive meta-analysis based on the most recent randomized controlled trials (RCTs) is needed to provide a multidimensional evaluation of gait, balance, and motor function, as well as to clarify protocol-specific stimulation effects. The present study was conducted to address these gaps in the existing evidence. Therefore, this study aimed to systematically evaluate RCTs that report the therapeutic effects of rTMS on gait, motor function, and balance in patients with PD and establish an objective rTMS protocol.

## 2. Method

### 2.1. Study Design

This systematic review and meta-analysis evaluated the effects of rTMS on balance and gait in patients with PD. This study was conducted in accordance with the Preferred Reporting Items for Systematic Reviews and Meta-Analyses (PRISMA) guidelines [27].

### 2.2. Literature Search and Study Selection

The literature search was conducted between November and December 2024. PubMed, EMBASE, Cochrane Central Register of Controlled Trials, Scopus, and Ovid-LWW were searched for relevant studies. Based on four primary keywords (“Parkinson”, “repetitive transcranial magnetic stimulation”, “Gait”, and “Balance”), related terms and synonyms were identified and combined to perform a comprehensive search. The complete search strategy, including Boolean search strings for each database, is presented in the Supplementary Materials (Table S1).

The studies identified through the database search were organized using EndNote 20 software, and duplicate records were removed. Additional duplicates that were not detected by the software were manually excluded using Microsoft Excel 365. Subsequently, three independent reviewers screened the titles and abstracts of the studies retrieved based on the eligibility criteria, excluding irrelevant articles. The full texts of the selected studies were then reviewed to confirm final inclusion. Selection reliability was assessed by calculating Cohen’s kappa coefficient. The inter-rater agreement for the study selection process was found to be excellent (k = 0.86). Any disagreements during the selection process were resolved through discussion with a third independent reviewer.

### 2.3. Eligibility Criteria

The inclusion criteria for study selection were as follows: (1) Studies were randomized controlled trials (RCTs). (2) Participants were diagnosed with Parkinson’s disease (PD). (3) Studies provided quantitative data on gait, balance, or motor function. (4) Studies were published in either English or Korean. (5) The full text was available.

The exclusion criteria were as follows: (1) Studies were non-experimental designs, such as observational studies, case reports, systematic reviews, meta-analyses, animal studies, or qualitative research. (2) Publications were conference abstracts or posters, or the studies lacked sufficient data for analysis. (3) The full text was not accessible. (4) Studies for which quantitative outcome data could not be extracted, even when results were presented in figures or tables. (5) Studies with evident data inconsistencies or reporting errors in tables or figures that could not be resolved after careful review.

### 2.4. Data Extraction and Quality Assessment

The following data were extracted from each study included in the analysis: first author’s name; publication year; study design; mean age; sex; disease duration; sample size; Hoehn and Yahr stage; assessment methods; outcomes related to gait; balance; and motor function; rTMS protocol; dropout rate; and whether intention-to-treat analysis was performed. All extracted data were recorded and managed using Microsoft Excel 365.

The methodological quality of the included studies was quantitatively assessed using the physiotherapy evidence database (PEDro) scale, which is a standardized tool for evaluating the quality of clinical trials. The PEDro scale consists of 11 items, each scored as either “yes” (1) or “no” (0) depending on whether the criterion is met. The total PEDro score was calculated by summing items 2–11, resulting in a score ranging from 0 to 10. Based on the total score, the study quality was classified into four levels: excellent (9–10), good (6–8), fair (4–5), and poor (≤4) [28]. Although the GRADE approach is widely recommended for assessing the certainty of evidence, we primarily utilized the PEDro scale as it offers a specific and rigorous assessment of internal validity (risk of bias) tailored for physical therapy and rehabilitation trials. To enhance the transparency of evidence reporting, the certainty of evidence was evaluated using the GRADE approach, and a Summary of Findings (SoF) table synthesizing the certainty of evidence was additionally provided.

### 2.5. Data Analysis and Synthesis

To calculate the effect sizes, the number of participants, means, and standard deviations from both the experimental and control groups were extracted. Outcomes reported as ranges (minimum–maximum) or medians with interquartile ranges (IQR) were converted into means and standard deviations for analysis, using the statistical algorithms described by Wan et al. [29]. All statistical analyses were performed using RStudio 2024.12.0ver.

The meta-analysis was conducted using Review Manager version 5.4. The inverse variance method was applied to calculate standardized mean differences (SMDs) for continuous outcomes [30]. Since the effect sizes may be overestimated in studies with small sample sizes, it is recommended to convert Cohen’s d to Hedges’ g [31]. Therefore, Hedges’ g was used as the effect size in this study. Statistical significance was determined by 95% confidence intervals (CIs) and *p*-values, and the heterogeneity of the results was evaluated based on the I^2^ statistic and chi-square tests (χ^2^).

To identify the preferred protocol of rTMS, subgroup analyses were conducted to compare the effects of specific parameters. The subgroup comparisons included stimulation frequency, target area, total pulses, session duration, number of sessions, and total treatment duration.

To visually assess small-study effects and publication bias, funnel plots were generated using RStudio 2024.12.0ver. A funnel plot, a scatterplot of effect estimates against sample size, is useful for detecting potential bias in meta-analyses [32]. In addition, Egger’s regression test was performed to statistically evaluate the presence of publication bias. A *p*-value < 0.05 indicated significant publication bias [33]. The PRISMA 2020 checklist is enclosed in the Supplementary Material (Figure S1) [34]. The review protocol was not registered in PROSPERO because data collection and analysis had been completed prior to the decision to register; therefore, registration was deemed not applicable.

## 3. Results

**Figure 1:**
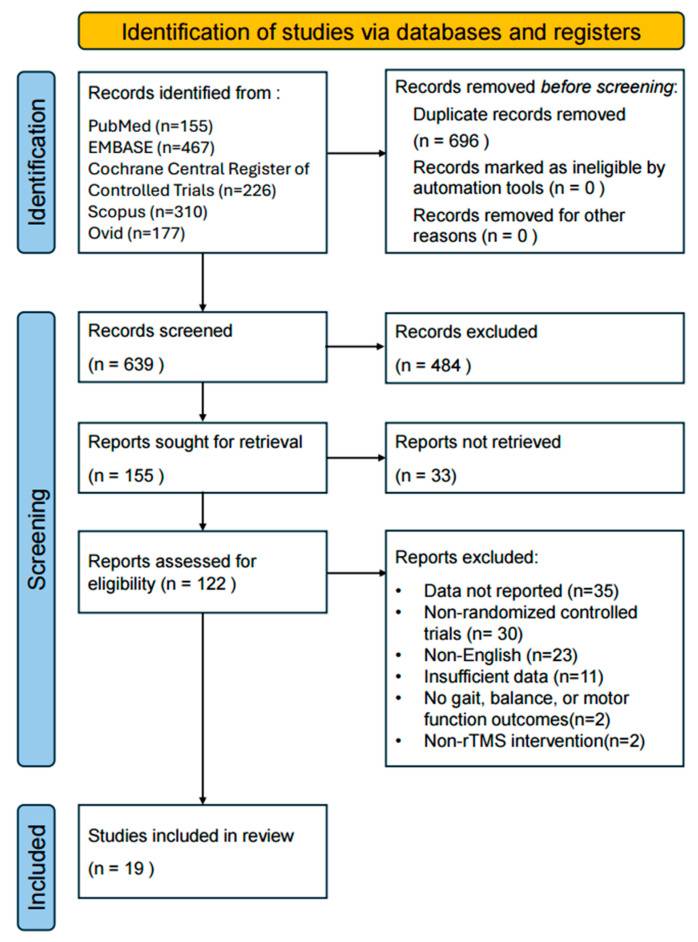
Flow chart diagram of study selection.

A total of 1335 articles were initially identified through database searches, which included 155 articles from PubMed, 467 from Embase, 310 from Scopus, 226 from Cochrane, and 177 from Ovid. After removing 696 duplicates using EndNote 20, 639 records remained. Based on a review of the titles and abstracts, 484 articles were excluded for not meeting the inclusion criteria. The full texts of the remaining 155 articles were assessed for eligibility according to the exclusion criteria. As a result, 19 studies were finally included in the systematic review and meta-analysis (Table 1 and Table 2, Figure 1).

### 3.1. Freezing of Gait Questionnaire (FOG-Q)

**Figure 2:**
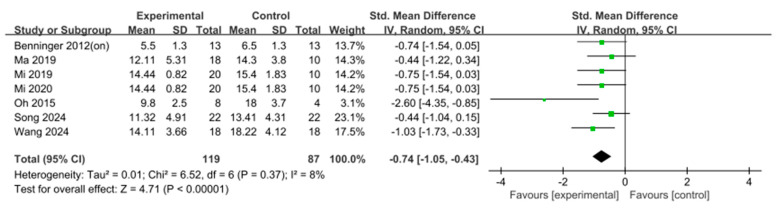
Forest plot of Freezing of Gait Questionnaire. Studies included: Benninger (2012) [36], Ma (2019) [44], Mi (2019) [26], Mi 2020 [45], Oh (2015) [46], Song (2024) [48], Wang (2024) [49].

A total of seven studies (*n* = 206) were included in the analysis. The results showed a significant effect (SMD = −0.74, 95% confidence interval [CI] = [−1.05, −0.43], I^2^ = 8%, Z = 4.71, *p* < 0.001). Because the number of studies included in this analysis was <10, statistical tests and visual assessments using funnel plots were not performed to evaluate publication bias (Figure 2).

### 3.2. Gait Speed

**Figure 3:**
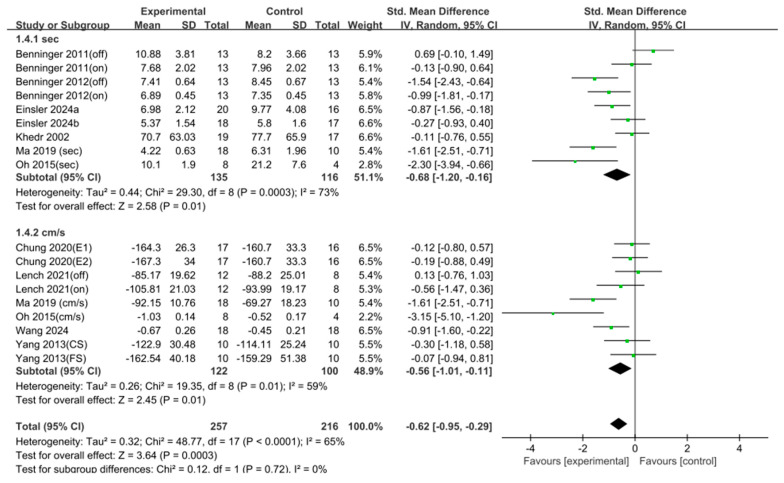
Forest plot of gait speed. Studies included: Benninger (2011) [35], Benninger (2012) [36], Chung (2020) [37], Einsler (2024a) [38], Einsler (2024b) [39], Khedr (2002) [40], Lench (2021) [41], Ma (2019) [44], Oh (2015) [46], Wang (2024) [49], Yang (2013) [50].

**Figure 4:**
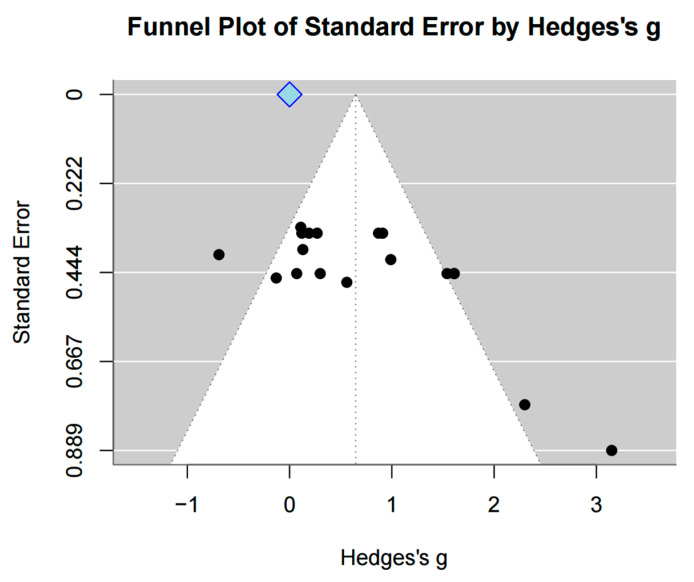
Funnel plot of gait speed.

Two different measurement approaches were used to assess gait speed. In the first method, the time (in seconds) required to walk a fixed distance was calculated. Seven studies (*n* = 251) were included in this analysis, and a significant effect was observed (SMD = 0.68, 95% CI = [0.16, 1.20], I^2^ = 73%, Z = 2.58, *p* = 0.01). In the second method, velocity was calculated as the distance walked per unit of time. Six studies (*n* = 222) were included, and a significant effect was also found (SMD = 0.56, 95% CI = [0.11, 1.01], I^2^ = 59%, Z = 2.45, *p* = 0.01). When both types of measurements were pooled, 13 studies (*n* = 473) were included in the analysis, revealing a significant overall effect (SMD = 0.62, 95% CI = [0.29, 0.95], I^2^ = 65%, Z = 3.64, *p* < 0.001). Egger’s regression test was used to statistically evaluate the asymmetry of the funnel plot (t = 2.69, df = 16). Significant publication bias was found (*p* = 0.016, 95% CI [−2.74, 0.20]). Duval and Tweedie’s trim-and-fill method was then employed. As a result, no imputed studies were performed; therefore, no change in effect size was noted (Figure 3 and Figure 4).

To further explore the differential effects of rTMS on gait speed, subgroup analyses were performed based on protocol characteristics, including stimulation frequency, target area, total pulses, session duration, number of sessions, and total treatment duration.

Frequency: High-frequency rTMS protocols (eight studies, *N* = 329) yielded a significant improvement in gait speed (SMD = 0.76, *p* = 0.001), whereas low-frequency protocols (three studies, *N* = 109) did not reach significance (SMD = 0.41, *p* = 0.07).

Target area: Among targeted regions, only the stimulation on the supplementary motor area (SMA; two studies, *N* = 96) produced a significant effect (SMD = 0.91, *p* = 0.03). Protocols targeting the primary motor cortex (M1; six studies, *N* = 282), dorsolateral prefrontal cortex (DLPFC; two studies, *N* = 76), and cerebellum (two studies, *N* = 71) demonstrated nonsignificant effects (M1, SMD = 0.35, *p* = 0.06; DLPFC, SMD = 0.99, *p* = 0.19; cerebellum, SMD = 0.56, *p* = 0.06).

Total pulses: Neither low-dose (≤1000 pulses; four studies, *N* = 179, SMD = 0.62, *p* = 0.08) nor high-dose (>1000 pulses; five studies, *N* = 206, SMD = 0.40, *p* = 0.07) protocols demonstrated significant effects.

Session duration: All session-length categories showed significant gains: ≥20 min (two studies, *N* = 96, SMD = 0.91, *p* = 0.03), <20 min (three studies, *N* = 111, SMD = 0.43, *p* = 0.03), and unspecified durations (five studies, *N* = 230, SMD = 0.61, *p* = 0.03).

Number of sessions: A regimen of 10 sessions (six studies, *N* = 227) yielded a robust effect (SMD = 0.93, *p* < 0.001), whereas alternative session counts (five studies, *N* = 246) did not achieve significance (SMD = 0.38, *p* = 0.07).

Total treatment duration: Short-term (<2 weeks; six studies, *N* = 171, SMD = 0.68, *p* = 0.02) and longer-term (≥2 weeks; five studies, *N* = 302, SMD = 0.59, *p* = 0.007) interventions produced significant improvements in gait speed (Table 3).

### 3.3. Unified Parkinson’s Disease Rating Scale III (UPDRS-III)

**Figure 5:**
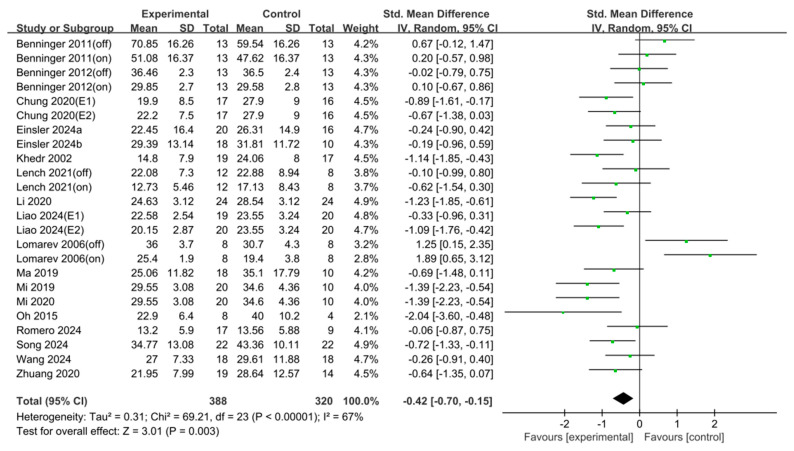
Forest plot of Unified Parkinson’s Disease Rating Scale Ⅲ. Studies included: Benninger (2011) [35], Benninger (2012) [36], Chung (2020) [37], Einsler (2024a) [38], Einsler (2024b) [39], Khedr (2002) [40], Lench (2021) [41], Li (2020) [42], Liao (2024) [25], Lomarev (2006) [43], Ma (2019) [44], Mi (2019) [26], Mi (2020) [45], Oh (2015) [46], Romero (2024) [47], Song (2024) [48], Wang (2024) [49], Zhuang (2020) [51].

**Figure 6:**
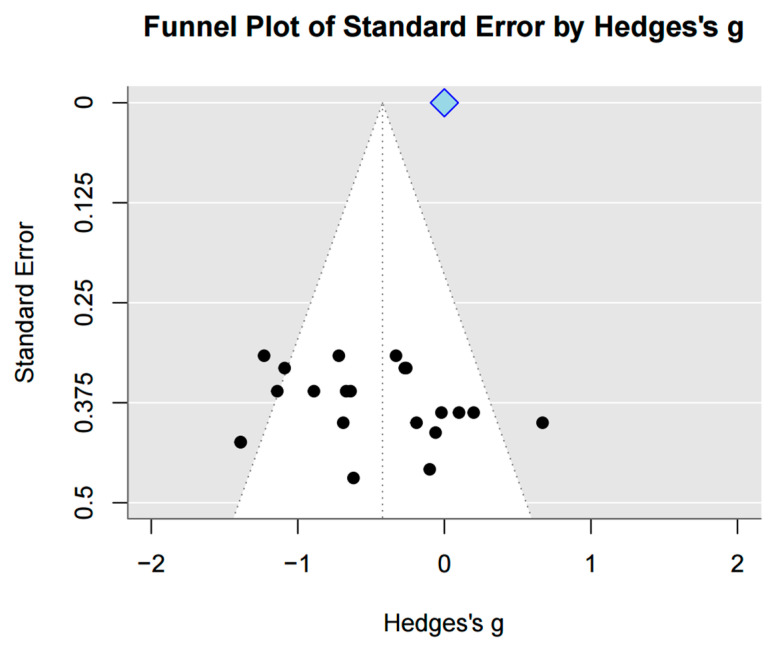
Funnel plot of Unified Parkinson’s Disease Rating Scale Ⅲ.

Analysis of 18 studies (*n* = 708) revealed a significant effect (SMD = −0.42, 95% CI [−0.70, −0.15], I^2^ = 67%, Z = 3.01, *p* = 0.003). Egger’s regression test for funnel plot asymmetry (t = 1.42, df = 22) did not detect significant publication bias (*p* = 0.169, 95% CI [–3.13, 0.04]). Consequently, Duval and Tweedie’s trim-and-fill method was not applied (Figure 5 and Figure 6).

To further investigate the specific effects of rTMS on UPDRS-III, subgroup analyses were performed based on rTMS protocol characteristics. The subgroup comparisons included stimulation frequency, target area, total pulses, session duration, number of sessions, and total treatment duration.

Frequency: High-frequency rTMS (13 studies, *n* = 461) showed no significant effect (SMD = −0.38, *p* = 0.07), whereas low-frequency rTMS (five studies, *n* = 221) did (SMD = −0.54, *p* < 0.001).

Target area: Significant improvements were seen with M1 (eight studies, *n* = 360, SMD = −0.39, *p* = 0.03) and SMA (five studies, *n* = 207, SMD = −0.79, *p* < 0.001) stimulations but not with DLPFC (four studies, *n* = 129, SMD = 0.27, *p* = 0.55) or cerebellar targets (two studies, *n* = 64, SMD = −0.22, *p* = 0.40).

Total pulses: In this study, >1000 pulses (seven studies, *n* = 314) yielded a significant effect (SMD = −0.81, *p* < 0.001), whereas ≤1000 pulses (eight studies, *n* = 278) did not (SMD = −0.11, *p* = 0.69).

Session duration: Sessions ≥ 20 min (eight studies, *n* = 332) were effective (SMD = −0.82, *p* < 0.001). Conversely, sessions < 20 min (two studies, *n* = 64) and those with unreported durations (nine studies, *n* = 340) were not.

Number of sessions: Exactly 10 sessions (10 studies, *n* = 317) produced a significant effect (SMD = −0.75, *p* < 0.001), whereas other dosing schedules (seven studies, *n* = 312) did not.

Total treatment duration: Both <2 weeks (eight studies, *n* = 277, SMD = −0.70, *p* < 0.001) and ≥2 weeks (eight studies, *n* = 320, SMD = −0.39, *p* = 0.04) of treatment duration showed significant improvements (Table 4).

### 3.4. Unified Parkinson’s Disease Rating Scale Total

**Figure 7:**
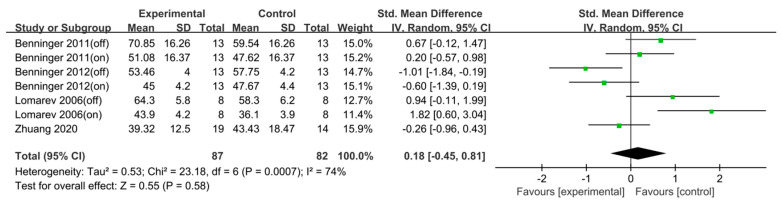
Forest plot of Unified Parkinson’s Disease Rating Scale total. Studies included: Benninger (2011) [35], Benninger (2012) [36], Lomarev (2006) [43], Zhuang (2020) [51].

Analysis of four studies (*n* = 169) revealed no significant effect (SMD = 0.18, 95% CI [−0.45, 0.81], I^2^ = 74%, Z = 0.55, *p* = 0.58). Given that the number of studies included was <10, statistical tests and visual assessment using funnel plots were not performed to evaluate publication bias (Figure 7).

### 3.5. Timed Up and Go (TUG)

**Figure 8:**
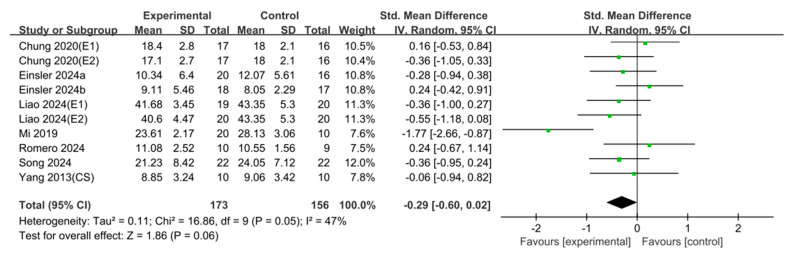
Forest plot of Timed up and Go test. Studies included: Chung (2020) [37], Einsler (2024a) [38], Einsler (2024b) [39], Liao (2024) [25], Mi (2019) [26], Romero (2024) [47], Song (2024) [48], Yang (2013) [50].

Analysis of eight studies (*n* = 329) showed no significant effect (SMD = −0.29, 95% CI [−0.60, 0.02], I^2^ = 47%, Z = 1.86, *p* = 0.06) (Figure 8).

The Summary of Findings (SoF) table summarizes the certainty of the evidence for the main outcome measures, according to the GRADE approach (Table 5).

## 4. Discussion

In this study, a systematic review and meta-analysis including only RCTs was conducted to comprehensively analyze the effects of rTMS on gait, motor function, and balance in patients with PD. A total of 19 RCTs involving 547 individuals with PD were included in the analysis. The meta-analysis revealed significant and moderate improvements in gait freezing (FOG-Q), gait speed, and motor symptoms (UPDRS-III) following rTMS intervention. However, no significant benefits were observed for UPDRS total scores or balance outcomes.

An increase in gait speed is clinically important because it is typically associated with an increase in step length [52]. This is particularly relevant in PD, as reduced step length is a key feature of hypokinetic gait [53]. Improving gait speed can lead to greater mobility, independence, and overall quality of life in individuals with PD [54].

Among the study outcomes, the effect size for the FOG-Q was −0.74, with a 95% CI of [−1.05, −0.43], indicating a significant difference. This finding is consistent with the results of Xie et al. [55], who also reported improvements in FOG-Q scores in the rTMS intervention group compared with the control group following treatment.

In the early stages of research, the focus was primarily on local synaptic plasticity [56,57]. However, recent network-level models offer a more nuanced explanation for the distinct effects of rTMS protocols observed in our subgroup analyses. As demonstrated in the relevant literature, there is an increasing body of evidence to suggest that Parkinsonian motor deficits, particularly those related to freezing of gait (FOG) and bradykinesia, are increasingly linked to pathological exaggerated beta-band (13–30 Hz) oscillations within the basal ganglia–thalamocortical circuits. These oscillations have been shown to act as an “anti-kinetic” state, which in effect prevents movement initiation [58,59].

The present study found that high-frequency (HF) repetitive transcranial magnetic stimulation (rTMS) over the superior motor area (SMA) optimally improves gait speed, which is in alignment with current theories on gait automaticity. In PD, the SMA, a pivotal node for both internal cueing and automatic movement control, has been observed to exhibit hypoactivity and reduced connectivity with the putamen [60,61]. It is hypothesized that high-frequency (HF) stimulation may enhance cortical excitability, thus normalizing SMA activity and restoring the “automatic” gait network that is bypassed in favor of slower, attention-demanding cerebellar compensation loops [62]. This restoration of SMA-striatal functional connectivity may provide a rationale for the superiority of HF-SMA protocols in achieving dynamic gait outcomes, as evidenced by our analysis.

Conversely, the present findings suggest that low-frequency (LF) stimulation is more efficacious than M1 and SMA for general motor symptoms (UPDRS-III), which can be interpreted through the normalization of cortical inhibition. The condition known as PD is characterized by impaired intracortical inhibition and cortical hyperexcitability in the primary motor cortex. These phenomena are attributable to thalamocortical dysrhythmia. Low-frequency repetitive transcranial magnetic stimulation (rTMS) has been demonstrated to suppress cortical excitability, with the potential to reduce this pathological noise and disrupt hypersynchronous beta activity [63]. This, in turn, may enhance the signal-to-noise ratio for voluntary motor commands. The dissociation of HF-SMA for gait automaticity versus LF-M1/SMA for reducing cortical hyperexcitability emphasizes the necessity of tailoring protocols to specific pathophysiological targets.

Regarding gait speed, two measurement approaches were used in the analysis: one based on the time required to walk a fixed distance and the other based on calculated velocity. The effect sizes for these two approaches were 0.68 and 0.56, with 95% CIs of [0.16, 1.20] and [0.11, 1.01], respectively, both indicating significant effects. When these results were pooled, the overall effect size was 0.62, with a 95% CI of [0.29, 0.95], again showing a significant effect.

Egger’s regression test indicated the presence of publication bias. However, when the trim-and-fill method was utilized to correct this bias, it identified no studies that needed imputation, suggesting that adjustment to the effect size was unnecessary.

A comparative study analyzing the effects of transcranial direct current stimulation (tDCS) and rTMS reported that rTMS did not produce a significant improvement in gait speed [64]. This contrasts with the result of the present study, which found a significant effect on gait speed. Although publication bias was detected in our analysis and the effect size should ideally be interpreted with caution or adjusted, the overall trend in previous studies still implies a potentially positive effect. Therefore, further research may yield more consistent and favorable results.

In a study based on magnetic resonance imaging applying 25 Hz rTMS to bilateral M1, relative changes were found in the functional connectivity between the SMA and prefrontal area during the performance of complex motor tasks [65]. The SMA, located anterior to the M1 region, is crucial in various motor processes and is activated before movement initiation [66]. Kim et al. [67] suggested that in patients with PD experiencing FOG, the SMA may be a more suitable target for brain stimulation than M1. However, when compared with the subgroup analysis on gait speed in the present study, although the frequency (high-frequency [HF] stimulation) aligns with prior findings, only two studies applied rTMS to the SMA, and the results were not significant. Therefore, interpretations should be made with caution.

Similarly, del Olmo et al. [68] reported that rTMS applied to the DLPFC yielded beneficial effects on gait in patients with PD but had no significant influence on motor task performance. In the present study, only two studies targeting the DLPFC were included in the subgroup analysis of gait speed, which limits interpretability. However, the effect sizes noted for DLPFC and SMA were among the highest observed, indicating that additional research could reveal more consistent and clinically meaningful benefits associated with these regions. Our subgroup analysis highlighted that high-frequency (HF) stimulation and targeting the SMA were the most effective protocols for improving gait speed. Regarding dosing, a regimen of exactly 10 sessions with durations exceeding 20 min yielded the most robust outcomes. Both short-term (<2 weeks) and long-term (≥2 weeks) interventions proved effective, suggesting flexibility in treatment duration. For the UPDRS-III outcome, the effect size was −0.42 with a 95% CI of [−0.70, −0.15], indicating a significant difference. Egger’s regression test was performed to assess publication bias, and no evidence of publication bias was found.

The UPDRS-III is a reliable and valid tool for assessing motor symptoms in individuals with PD and correlates with disease severity and quality of life [69,70]. In contrast to gait speed, UPDRS-III scores improved most significantly with Low-Frequency (LF) stimulation. While both M1 and SMA targets were effective, SMA stimulation demonstrated a larger effect size. Additionally, protocols utilizing a higher total pulse count (>1000 pulses) and longer session durations (≥20 min) were associated with greater motor symptom relief. Interestingly, shorter intervention periods (<2 weeks) appeared to yield slightly more favorable results than longer durations.

Previous studies have supported these findings. Strafella et al. [71] reported that rTMS applied to M1 activates corticostriatal projections, which facilitates dopamine release in the striatum, thereby positively affecting motor function in PD. Regarding the SMA, Koch et al. [72] reported that LF (1 Hz) rTMS significantly reduced levodopa-induced dyskinesia, whereas HF (5 Hz) rTMS produced a mild but nonsignificant increase. Brusa et al. [73] also observed that rTMS can induce temporary reductions in dyskinesia but may not be clinically useful.

Although these prior studies predominantly employed LF stimulation, which showed significant effects in the subgroup analysis of the present study, noted that only five studies examined LF stimulation. This limited number, particularly compared with the greater number of HF studies, warrants cautious interpretation. Given that HF rTMS did not demonstrate a significant effect in our analysis, these findings are in line with the present results.

For the UPDRS total, the effect size was 0.18 with a 95% CI of [−0.42, 0.81], indicating no significant difference.

The use of rTMS to treat motor symptoms in PD has been relatively underexplored, and studies reporting improvements in motor symptoms following rTMS intervention over the right DLPFC remain limited and inconsistent [74]. In the present study, only four such studies were included, making it difficult to draw definitive conclusions regarding the efficacy of rTMS on motor symptoms. Therefore, future studies are recommended to further investigate this area, allowing us to establish more meaningful and conclusive evidence through the accumulation of well-designed studies.

In the analysis of balance-related outcomes using the TUG test, the effect size was −0.29 with a 95% CI of [−0.60, 0.02], indicating no significant difference. The TUG test consists of multiple components, including gait initiation, straight-line walking, and turning, each requiring varying levels of cognitive control [75]. Although straight walking generally demands the least cognitive effort, turning requires significantly more cognitive regulation to adjust gait patterns [76], particularly due to the need for dynamic balance control during directional changes [77]. Although the results of this study did not show a significant improvement in balance, the observed trend was positive. Therefore, future studies with larger sample sizes and refined methodologies may reveal more meaningful and conclusive outcomes.

### 4.1. Interpretation of the PEDro Score and Publication Bias

In this meta-analysis, participants in all 19 included studies were randomly assigned to either experimental or control groups. The included studies demonstrated high methodological quality, with an average PEDro score of 7.73, which is notably higher than the average score of 5.0 typically reported in physiotherapy trials [78]. Given that statistical tests for publication bias did not reveal significant evidence in most analyzed, the internal validity of these findings is considered robust. The average score in this review was notably higher than the average PEDro score of 5 reported in the PEDro database [78].

Given that statistical tests for publication bias did not reveal significant evidence of bias in most of the analyses, the internal validity of this meta-analysis is inferred to be sufficiently assured. Egger’s regression has limited statistical power in the analysis with a small number of studies included [79]. Therefore, additional sensitivity analyses, such as the trim-and-fill method, were employed and presented in this study. However, the trim-and-fill method assumes symmetry in the funnel plot and does not account for potential causes of funnel plot asymmetry [80], nor does it reveal the underlying mechanisms of publication bias [81]. In this study, Egger’s regression and funnel plot analysis detected publication bias and funnel plot asymmetry for the gait speed outcome. Accordingly, interpretation of the results derived from the trim-and-fill method for this outcome should be considered with caution.

### 4.2. Study Limitations

This study has several notable limitations: First, the number of included studies was relatively small, which limits the generalizability of the findings. Second, despite systematic electronic database searches, publication bias cannot be completely ruled out, and subjective judgment in data assessment may have introduced observer bias. Third, several important potential confounders were not fully controlled due to data limitations. The observed discrepancies in motor outcomes could be attributed to variations in medication state (ON vs. OFF) and disease severity heterogeneity across studies. Additionally, the term motor reserve was not included in the study, despite its growing recognition as a significant modifier of motor outcomes in PD [82,83]. Motor reserve is defined as the capacity of the motor system to cope with PD-related pathology, thereby explaining why some patients maintain better motor function than others despite similar levels of neurodegeneration. The presence of variability in motor reserve among participants has the potential to influence their responsiveness to rTMS, thereby contributing to the observed heterogeneity in therapeutic effects. Similarly, the presence of depressive symptoms could not be fully controlled for across all the included studies. Depression is a prevalent non-motor symptom in PD, with significant implications for motor function, disability, and response to treatment [84]. Although some of the included trials assessed outcomes related to mood, the potential confounding effect of baseline depression on rTMS-induced motor improvements was not systematically analyzed. Furthermore, methodological discrepancies, including stimulation laterality (unilateral vs. bilateral) and coil type, along with the magnitude of placebo effects, were not subjected to individual stratification during the interpretation of results. These factors may constrain the strength of our clinical recommendations and should be carefully considered in future trials. Lastly, the included studies employed heterogeneous rTMS protocols and differed in treatment duration, which may have introduced bias into the results. Another significant limitation is that this review protocol was not preregistered in PROSPERO. The absence of a preregistered protocol has been demonstrated to engender a lack of methodological transparency and to introduce the potential for selective reporting bias. In the absence of a publicly recorded plan prior to data analysis, there is an inherent risk that certain methodological decisions including specific inclusion criteria or the selection of subgroup analyses could have been influenced by post hoc considerations. Despite our efforts to minimize this bias through strict adherence to the PRISMA 2020 guidelines during the reporting process, the absence of preregistration remains a constraint on the reproducibility and impartiality of our findings. Future studies that address these limitations may help provide more robust evidence regarding the effectiveness of rTMS interventions in individuals with PD.

## 5. Conclusions

In this study, a systematic review and meta-analysis was conducted to comprehensively analyze changes in gait, motor function, and balance when rTMS was applied to patients with PD. A total of 19 RCTs were selected and analyzed, confirming significant effects on FOG-Q, gait speed, and UPDRS Part III. The integrated results indicate that high-frequency (HF) stimulation targeting the supplementary motor area (SMA) appears to yield beneficial outcomes for gait speed. With regard to UPDRS-III, the stimulation of both the M1 and SMA with low-frequency (LF) rTMS has been demonstrated to be effective, particularly when utilizing protocols involving >1000 pulses and sessions lasting ≥20 min. However, given the limited number of studies included in some subgroups (particularly for the SMA), these findings regarding optimal protocols should be interpreted with caution. It is evident that further large scale randomized controlled trials are required in order to validate these parameters. Only upon validation can they be established as standard clinical guidelines.

## Figures and Tables

**Table 1:** The Main Characteristics of the Studies Included in the Meta-analysis.

Study (Year)	Age (Year, Mean SD)	Sex (M/F)	Disease Duration (Year, Mean ± SD)	Hoehn and Yahr (Score, Mean ± SD)	Outcomes	PEDro Score
Benninger (2011) [35]	^1^ E: 62.1 ± 6.9, ^2^ C: 65.6 ± 9.0	E: 7/6, C: 11/2	E: 10.8 ± 7.1 C: 6.5 ± 3.4	E(on): 2.6 ± 0.2 C: 2.5 ± 0.1	gait speed, ^6^ UPDRSⅢ, ^6^ UPDRS_total	8
Benninger (2012) [36]	E(on): 64.5 ± 9.1, C(on): 63.7 ± 8.3	E: 11/2, C: 9/4	E: 8.6 ± 4.1 C: 9.3 ± 6.8	E(on): 2.4 ± 0.2 (off): 2.7 ± 0.3 C(on): 2.5 ± 0.3 (off): 2.9 ± 0.6	^5^ FOG-Q, ^4^ 10MWT, ^6^ UPDRSⅢ, ^6^ UPDRS_total	8
Chung (2020) [37]	E1: 62.7 ± 6.8, E2: 62.1 ± 5.7, C: 62.1 ± 5.7	E1: 10/7 E2: 9/8 C: 7/9	E1: 5.2 ± 3.4 E2: 7.5 ± 4.9 C: 6.9 ± 3.3	E1: 2.2 ± 0.3 E2: 2.2 ± 0.4 C: 2.3 ± 0.3	gait speed, ^6^ UPDRSⅢ, ^7^ TUG	9
Einsler (2024a) [38]	E: 68 (61–77) C: 71 (66–77)	E: 14/6 C: 10/6	-	E: 2.0 (1–3) C: 1.5 (1–3)	^3^ 8MWT, ^6^ UPDRSⅢ, ^7^ TUG	10
Einsler (2024b) [39]	E: 66.06 ± 9.70 C: 70.41 ± 10.37	E: 15/3 C: 13/4	-	E: 2.11 ± 0.9 C: 1.94 ± 0.75	^3^ 8MWT, ^6^ UPDRSⅢ, ^7^ TUG	8
Khedr (2002) [40]	E: 57.8 ± 9.2 C: 57.5 ± 8.4	E: 14/5 C: 10/7	E: 3.05 ± 2.1 C: 3.6 ± 2.4	-	gait speed, ^6^ UPDRSⅢ	6
Lench (2021) [41]	E: 66.6 ± 7.5 C: 64.5 ± 8.9	E: 7/5 C: 7/1	E: 8.7 ± 7.12 C: 8 ± 5.63	E: 2.32 ± 0.405 C: 2.29 ± 0.267	gait speed, ^6^ UPDRSⅢ	8
Li (2020) [42]	E: 61.67 ± 6.92 C: 61.46 ± 8.4	E: 8/16 C: 8/16	E: 5.48 ± 3.69 C: 6.46 ± 5.17	E: 1.85 ± 0.63 C: 1.83 ± 0.64	^6^ UPDRSⅢ	8
Liao (2024) [25]	E1: 64.89 ± 9.31 E2: 62.58 ± 10.34 C: 59.95 ± 10.66	E1: 6/13 E2: 8/12 C: 10/10	E1: 7.21 ± 3.51 E2: 6.95 ± 2.54 C: 6.45 ± 2.61	E1: 2.74 ± 0.87 E2: 2.55 ± 1.05 C: 2.75 ± 1.02	^6^ UPDRSⅢ, ^7^ TUG	8
Lomarev (2006) [43]	E: 63 ± 10 C: 66 ± 10	E: 7/2 C: 8/1	E: 13.8 ± 6.8 C: 10.8 ± 3.1	-	^6^ UPDRSⅢ, ^6^ UPDRS_total	4
Ma (2019) [44]	E: 59.94 ± 9.16 C: 66 ± 8.55	E: 8/10 C: 5/5	E: 8.94 ± 5.48 C: 7.5 ± 4.72	E: 2.42 ± 0.6 C: 2.4 ± 0.94	^5^ FOG-Q, ^6^ UPDRSⅢ, gait time, gait velocity	10
Mi (2019) [26]	E: 62.65 ± 10.56 C: 65.60 ± 8.68	E: 9/11 C: 5/5	E: 9.15 ± 5.82 C: 7.40 ± 4.83	E: 2.60 ± 0.85 C: 2.35 ± 0.91	^5^ FOG-Q, ^6^ UPDRSⅢ, ^7^ TUG	9
Mi (2020) [45]	E: 62.65 ± 10.56 C: 65.60 ± 8.68	E: 9/11 C: 5/5	E: 9.15 ± 5.82 C: 7.40 ± 4.83	E: 2.6 ± 0.85 C: 2.35 ± 0.91	^5^ FOG-Q, ^6^ UPDRSⅢ	7
Oh (2015) [46]	E: 69.8 ± 8.2 C: 73.3 ± 1.9	E: 7/1 C: 1/3	E: 76.1 ± 37.1 (month) C: 64.3 ± 19 (month)	E: 3 ± 0 C: 3.25 ± 0.5	^5^ FOG-Q, ^6^ UPDRSⅢ, gait time, gait velocity	6
Romero (2024) [47]	E: 64.40 ± 6.38 C: 66.89 ± 9.07	E: 7/3 C: 6/3	E: 6.00 ± 3.06 C: 6.22 ± 4.12	E: 2.0 ± 0.52 C: 1.83 ± 0.5	^6^ UPDRSⅢ, ^7^ TUG	7
Song (2024) [48]	E: 67.36 ± 6.99 C: 70.50 ± 6.76	E: 15/7 C: 13/7	E: 6.18 ± 1.62 C: 6.77 ± 2.02	E: (2–2.5): 15, (3): 7 C: (2–2.5): 14, (3): 8	^5^ FOG-Q, ^6^ UPDRSⅢ, ^7^ TUG	8
Wang (2024) [49]	E: 70.33 ± 9.36 C: 73.39 ± 6.15	E: 8/10 C: 10/8	E: 7(5–10) C: 5.5(3–8.25)	E: 3 (2.38–3) C: 3 (2.5–3)	^5^ FOG-Q, ^6^ UPDRSⅢ, gait speed	8
Yang (2013) [50]	E: 65.20 ± 11.08 C: 67.00 ± 13.21	E: 5/5 C: 7/3	E: 6.40 ± 2.76 C: 6.35 ± 3.58	E: 2.30 ± 0.42 C: 2.35 ± 0.41	^4^ 10MWT, ^7^ TUG	7
Zhuang (2020) [51]	E: 60.58 ± 9.21 C: 61.57 ± 13.25	E: 11/8 C: 7/7	E: 70.37 ± 52.26 (month) C: 68.57 ± 45.29 (month)	E: 2 (1.5–2.5) C: 2.25 (1.75–3.0)	^6^ UPDRSⅢ, ^6^ UPDRS_total	8

^1^ E: experimental group; ^2^ C: control group; ^3^ 8MWT: 8 m walking test; ^4^ 10MWT: 10 m walking test; ^5^ FOG-Q: Freezing of Gait Questionnaire; ^6^ UPDRS: Unified Parkinson’s Disease Rating Scale; ^7^ TUG: Timed Up and Go test.

**Table 2:** rTMS Stimulation Parameters and Outcome Measures of the Included Studies.

Study (Year)	Mode (Frequency)	Target Area	Pulses	Number of Session	Total Duration	Status	Sample Size
Benninger (2011) [35]	^1^ iTBS (50 Hz)	M1, ^5^ DLPFC	600	8	2 weeks	on & off	26
Benninger (2012) [36]	^3^ HF (50 Hz)	M1	-	8	2 weeks	on & off	26
Chung (2020) [37]	^7^ E1:^3^ HF (25 Hz) ^8^ E2:^4^ LF (1 Hz)	M1	1200	12	3 weeks	on	50
Einsler (2024a) [38]	^1^ iTBS (48 Hz)	cerebellum	-	15	5 days	on	36
Einsler (2024b) [39]	^2^ TBS (48 Hz)	cerebellum	-	10	5 days	on	35
Khedr (2002) [40]	^3^ HF (5 Hz)	M1	2000	10	10 days	on	36
Lench (2021) [41]	^4^ LF (1 Hz)	^6^ SMA	1200	10	10 days	on & off	20
Li (2020) [42]	^3^ HF (20 Hz)	M1	2000	5	5 days	on	48
Liao (2024) [25]	^4^ LF (0.5 Hz)	^6^ SMA	1800	-	-	-	59
Lomarev (2006) [43]	^3^ HF (25 Hz)	M1, ^5^ DLPFC	300	8	4 weeks	on & off	16
Ma (2019) [44]	^3^ HF (10 Hz)	^6^ SMA	1000	10	2 weeks	on	28
Mi (2020) [45]	^3^ HF (10 Hz)	^6^ SMA	1000	10	2 weeks	on	30
Oh (2015) [46]	^3^ HF (5 Hz)	M1, ^5^ DLPFC	1200	10	10 days	on & off	12
Romero (2024) [47]	^3^ HF (10 Hz)	M1	1000	8	2 weeks	on	19
Song (2024) [48]	^3^ HF (10 Hz)	M1	1000	10	10 days	on	44
Wang (2024) [49]	^4^ LF (1 Hz)	M1	800	10	2 weeks	on	36
Yang (2013) [50]	^3^ HF (5 Hz)	M1	1200	12	4 weeks	on	20
Zhuang (2020) [51]	^4^ LF (1 Hz)	^5^ DLPFC	1200	10	10 days	off	33

^1^ iTBS: intermittent Theta Burst Stimulation; ^2^ TBS: Theta Burst Stimulation; ^3^ HF: High Frequency; ^4^ LF: Low Frequency; ^5^ DLPFC: DorsoLateral PreFrontal Cortex; ^6^ SMA: Supplementary Motor Area; ^7^ E1: Experiment group 1; ^8^ E2: Experiment group 2.

**Table 3:** Subgroup Analysis of Gait Speed.

Category	Subgroups	No. of Trials (Reference)	Sample Size	^3^ SMD(d)	95% CI	Heterogeneity *p* Value of Chi-Square Test (I^2^)	Overall Effect Z Value (*p* Value)
Frequency	^1^ HF	8	329	0.76	0.30, 1.22	43.61 (72%)	3.23 (0.001 *)
	^2^ LF	3	109	0.41	−0.03, 0.86	3.91 (23%)	1.81 (0.07)
Target area	M1	6	282	0.35	−0.01, 0.71	20.16 (55%)	1.9 (0.06)
	^4^ DLPFC	2	76	0.99	−0.47, 2.45	19.91 (85%)	1.32 (0.19)
	^5^ SMA	2	96	0.91	0.07, 1.75	10.44 (71%)	2.13 (0.03 *)
	Cerebellum	2	71	0.56	−0.03, 1.15	1.51 (34%)	1.87 (0.06)
Total pulses	≤1000 pulses	4	179	0.62	−0.06, 1.3	22.97 (78%)	1.78 (0.08)
	>1000 pulses	5	206	0.4	−0.03, 0.82	16.05 (50%)	1.83 (0.07)
Session duration	≥20 min	2	96	0.91	0.07, 1.75	10.44 (71%)	2.13 (0.03 *)
	<20 min	3	111	0.43	0.05, 0.81	2.83 (0%)	2.22 (0.03 *)
	Not report	5	230	0.61	0.05, 1.18	30.48 (74%)	2.13 (0.03 *)
Number of sessions	10 times	6	227	0.93	0.38, 1.47	26.08 (69%)	3.33 (0.000 **)
	< or >10 times	5	246	0.38	−0.03, 0.78	19.26 (58%)	1.84 (0.07)
Total treatment duration	<2 weeks	6	171	0.68	0.10, 1.25	16.57 (64%)	2.32 (0.02 *)
	≥2 weeks	5	302	0.59	0.16, 1.03	32.14 (69%)	2.69 (0.007 *)

^1^ HF: High Frequency; ^2^ LF: Low Frequency; ^3^ SMD: Standardized Mean Differences; ^4^ DLPFC: DorsoLateral PreFrontal Cortex; ^5^ SMA: Supplementary Motor Area; * *p* < 0.05; ** *p* < 0.001.

**Table 4:** Subgroup Analysis of UPDRSⅢ.

Category	Subgroups	No. of Trials (Reference)	Sample Size	^3^ SMD(d)	95% CI	Heterogeneity *p* Value of Chi-Square Test (I^2^)	Overall Effect Z Value (*p* Value)
Frequency	^1^ HF	13	461	−0.38	−0.79, 0.03	63.00 (76%)	1.82 (0.07)
	^2^ LF	5	221	−0.54	−0.82, −0.27	4.93 (0%)	3.91 (0.000 **)
Target area	M1	8	360	−0.39	−0.75, −0.04	27.12 (63%)	2.17 (0.03 *)
	^4^ DLPFC	4	129	0.27	−0.62, 1.15	25.35 (80%)	0.59 (0.55)
	^5^ SMA	5	207	−0.79	−1.16, −0.43	9.15 (34%)	4.23 (0.000 **)
	Cerebellum	2	64	−0.22	−0.72, 0.29	0.01 (0%)	0.85 (0.4)
Total pulses	≤1000 pulses	8	278	−0.11	−0.68, 0.45	42.66 (79%)	0.40 (0.69)
	>1000 pulses	7	314	−0.81	−1.08, −0.55	10.92 (18%)	6.10 (0.000 **)
Session duration	≥20 min	8	332	−0.82	−1.08, −0.56	11.24 (20%)	6.2 (0.000 **)
	<20 min	2	64	−0.23	−0.73, 0.27	0.02 (0%)	0.89 (0.37)
	Not report	9	340	−0.1	−0.52, 0.33	41.53 (71%)	0.45 (0.65)
Number of sessions	10 times	10	317	−0.75	−1.04, −0.46	14.53 (31%)	5.03 (0.000 **)
	< or >10 times	7	312	0.00	−0.47, 0.47	40.07 (75%)	0.01 (1.00)
Total treatment duration	<2 weeks	8	277	−0.7	−1.02, −0.38	12.49 (36%)	4.27 (0.000 **)
	≥2 weeks	8	320	−0.39	−0.76, −0.02	41.91 (55%)	2.05 (0.04 *)

^1^ HF: High Frequency; ^2^ LF: Low Frequency; ^3^ SMD: Standardized Mean Differences; ^4^ DLPFC: DorsoLateral PreFrontal Cortex; ^5^ SMA: Supplementary Motor Area; * *p* < 0.05; ** *p* < 0.001.

**Table 5:** Summary of Findings.

Outcome	No. of Participants (Studies)	Effect (95% CI) (^1^ SMD)	Certainty of the Evidence (^6^ GRADE)	Plain Language Summary
Freezing of Gait (^2^ FOG-Q)	206 (7 studies)	^1^ SMD −0.74 [−1.05 to −0.43]	⊕⊕⊕⊝ Moderate (Due to Imprecision ^a^)	^5^ rTMS likely reduces freezing of gait significantly.
Gait Speed	473 (13 studies)	^1^ SMD 0.62 [0.29 to 0.95]	⊕⊕⊝⊝ Low (Due to Inconsistency ^b^ and Publication bias ^c^)	^5^ rTMS may improve gait speed, but heterogeneity and publication bias exist.
Motor Symptoms (^3^ UPDRS-III)	708 (18 studies)	^1^ SMD −0.42 [−0.70 to −0.15]	⊕⊕⊕⊝ Moderate (Due to Inconsistency ^b^)	^5^ rTMS likely improves motor symptoms significantly.
Balance (^4^ TUG)	329 (8 studies)	^1^ SMD −0.29 [−0.60 to 0.02]	⊕⊕⊝⊝ Low (Due to Imprecision ^a^ and Inconsistency ^b^)	The effect of ^5^ rTMS on balance is uncertain (result not significant).

^a^ Downgraded one level due to imprecision, as the total sample size was relatively small and the confidence intervals were wide for the FOG-Q and TUG outcomes. ^b^ Downgraded one level due to substantial heterogeneity across studies (I^2^ > 50%). ^c^ Downgraded one level due to evidence of publication bias indicated by Egger’s regression test (*p* < 0.05). ^1^ SMD: Standardized Mean Differences. ^2^ FOG-Q: Freezing of Gait Questionnaire. ^3^ UPDRS: Unified Parkinson’s Disease Rating Scale. ^4^ TUG: Timed Up and Go test. ^5^ rTMS: Repetitive Transcranial Magnetic Stimulation. ^6^ GRADE: Grading of Recommendations Assessment, Development and Evaluation. ⊕⊕⊕⊝: moderate certainty of evidence; ⊕⊕⊝⊝: low certainty of evidence.

## Data Availability

The datasets generated during and/or analyzed during the current research are available from the corresponding author upon reasonable request.

## Supplementary Materials

The following supporting information can be downloaded at: https://www.mdpi.com/article/10.3390/jcm15010166/s1, Figure S1: PRISMA 2020 checklist; Table S1: Search strategy.
