# Supplementary material for: The Effects of Repetitive Transcranial Magnetic Stimulation on Gait, Motor Function, and Balance in Parkinson’s Disease: A Systematic Review and Meta-Analysis of Randomized Controlled Trials
Source: J Clin Med. 2025 Dec 25;15(1):166. doi: 10.3390/jcm15010166 (PMC12786542; doi:10.3390/jcm15010166)
Supplement: Supplementary file 1 [file jcm-15-00166-s001.zip › jcm-4010198-supplementary/Table S1. Search strategy.pdf]

Table S1. Search strategy

Database: PubMed

Search strategy:

#1 (((Parkinson disease[Title/Abstract]) OR (Parkinson's disease[Title/Abstract])) OR (PD[Title/Abstract])) OR (parkinsonism[Title/Abstract])

#2 ((((non-invasive brain stimulation[Title/Abstract]) OR (Transcranial Magnetic Stimulation[Title/Abstract])) OR (repetitive transcranial magnetic stimulation[Title/Abstract])) OR (TMS[Title/Abstract])) OR (rTMS[Title/Abstract])

#3 (((gait*[Title/Abstract]) OR (walk[Title/Abstract])) OR (walking[Title/Abstract])) OR (ambulation[Title/Abstract])

#4 balance*[Title/Abstract]

#5 ((#1) AND (#2)) AND (#3)

#6 ((#1) AND (#2)) AND (#4)

#7 (#5) OR (#6)

Database: Embase

Search strategy:

#1 'parkinson disease'/exp OR 'parkinson disease' OR 'parkinsonism'/exp OR 'parkinsonism'

#2 'non invasive brain stimulation'/exp OR 'non invasive brain stimulation' OR 'transcranial magnetic stimulation'/exp OR 'transcranial magnetic stimulation' OR 'repetitive transcranial magnetic stimulation'/exp OR 'repetitive transcranial magnetic stimulation'

#3 'gait'/exp OR 'gait' OR 'walking'/exp OR 'walking' OR 'mobilization'/exp OR 'mobilization'

#4 'balance disorder'/exp OR 'balance disorder' OR 'balance'/exp OR 'balance'

#5 #1 AND #2 AND #3

#6 #1 AND #2 AND #4

#7 #5 OR #6

Database: Cochrane Central Register of Controlled Trials

Search strategy:

#1 Parkinson disease OR Parkinson's disease OR PD OR parkinsonism

#2 non-invasive brain stimulation OR Transcranial Magnetic Stimulation OR repetitive transcranial magnetic stimulation OR TMS OR rTMS

#3 gait* OR walk OR walking OR ambulation

#4 balance*

#5 #1 AND #2 AND #3

#6 #1 AND #2 AND #4

#7 #5 OR #6

Database: Scopus

Search strategy:

(((TITLE-ABS-KEY ("parkinson disease") OR TITLE-ABS-KEY ("Parkinson's disease") OR TITLE-ABS-KEY (pd) OR TITLE-ABS-KEY (parkinsonism))) AND ((TITL(((TITLE-ABS-KEY ("parkinson disease") OR TITLE-ABS-KEY ("Parkinson's disease") OR TITLE-ABS-KEY (pd) OR TITLE-ABS-KEY (parkinsonism))) AND ((TITLE-ABS-KEY ("non invasive brain stimulation") OR TITLE-ABS-KEY ("Transcranial Magnetic Stimulation") OR TITLE-ABS-KEY ("repetitive Transcranial Magnetic Stimulation") OR TITLE-ABS-KEY (tms) OR TITLE-ABS-KEY (rtms))) AND ((TITLE-ABS-KEY (gait*) OR TITLE-ABS-KEY (walk) OR TITLE-ABS-KEY (walking) OR TITLE-ABS-KEY (ambulation)))) OR (((TITLE-ABS-KEY ("parkinson disease") OR TITLE-ABS-KEY ("Parkinson's disease") OR TITLE-ABS-KEY (pd) OR TITLE-ABS-KEY (parkinsonism))) AND ((TITLE-ABS-KEY ("non invasive brain stimulation") OR TITLE-ABS-KEY ("Transcranial Magnetic Stimulation") OR TITLE-ABS-KEY ("repetitive Transcranial Magnetic Stimulation") OR TITLE-ABS-KEY (tms) OR TITLE-ABS-KEY (rtms))) AND (TITLE-ABS-KEY (balance*)))E-ABS-KEY ("non invasive brain stimulation") OR TITLE-ABS-KEY ("Transcranial Magnetic Stimulation") OR TITLE-ABS-KEY ("repetitive Transcranial Magnetic Stimulation") OR TITLE-ABS-KEY (tms) OR TITLE-ABS-KEY (rtms))) AND ((TITLE-ABS-KEY (gait*) OR TITLE-ABS-KEY (walk) OR TITLE-ABS-KEY (walking) OR TITLE-ABS-KEY (ambulation))))) OR (((TITLE-ABS-KEY ("parkinson disease") OR TITLE-ABS-KEY ("Parkinson's disease") OR TITLE-ABS-KEY (pd) OR TITLE-ABS-KEY (parkinsonism))) AND ((TITLE-ABS-KEY ("non invasive brain stimulation") OR TITLE-ABS-KEY ("Transcranial Magnetic Stimulation") OR TITLE-ABS-KEY ("repetitive Transcranial Magnetic Stimulation") OR TITLE-ABS-KEY (tms) OR TITLE-ABS-KEY (rtms))) AND (TITLE-ABS-KEY (balance*)))

Database: Ovid-LWW

Search strategy:

#1 (Parkinson disease or Parkinson's disease or PD or parkinsonism).ab.

#2 (Parkinson disease or Parkinson's disease or PD or parkinsonism).ti.

#3 1 or 2

#4 (non-invasive brain stimulation or Transcranial Magnetic Stimulation or repetitive transcranial magnetic stimulation or TMS or rTMS).ti.

#5 (non-invasive brain stimulation or Transcranial Magnetic Stimulation or repetitive transcranial magnetic stimulation or TMS or rTMS).ab.

#6 4 or 5

#7 (gait* or walk or walking or ambulation).ab.

#8 (gait* or walk or walking or ambulation).ti.

#9 7 or 8

#10 balance*.ti.

#11 balance*.ab.

#12 10 or 11

#13 3 and 6 and 9

#14 3 and 6 and 12

#15 13 or 14
